# Prospective Memory and Regional Functional Connectivity in Subcortical Ischemic Vascular Disease

**DOI:** 10.3389/fnagi.2021.686040

**Published:** 2021-08-20

**Authors:** Xuan-Miao Zhuang, Li-Wei Kuo, Shih-Yen Lin, Jir-Jei Yang, Min-Chien Tu, Yen-Hsuan Hsu

**Affiliations:** ^1^Department of Psychology, National Chung Cheng University, Chiayi, Taiwan; ^2^Institute of Biomedical Engineering and Nanomedicine, National Health Research Institutes, Miaoli, Taiwan; ^3^Institute of Medical Device and Imaging, National Taiwan University College of Medicine, Taipei, Taiwan; ^4^Department of Computer Science, National Chiao Tung University, Hsinchu, Taiwan; ^5^Department of Medical Imaging, Taichung Tzu Chi Hospital, Buddhist Tzu Chi Medical Foundation, Taichung, Taiwan; ^6^Department of Neurology, Taichung Tzu Chi Hospital, Buddhist Tzu Chi Medical Foundation, Taichung, Taiwan; ^7^Department of Neurology, School of Medicine, Tzu Chi University, Hualien, Taiwan; ^8^Center for Innovative Research on Aging Society (CIRAS), National Chung Cheng University, Chiayi, Taiwan

**Keywords:** subcortical ischemic vascular disease, small vessel disease, vascular cognitive impairment, prospective memory, neuropsychological function, functional connectivity, regional homogeneity

## Abstract

**Objectives:** Patients with subcortical ischemic vascular disease (SIVD) often have prominent frontal dysfunction. However, it remains unclear how SIVD affects prospective memory (PM), which strongly relies on the frontoparietal network. The present study aimed to investigate PM performance in patients with early stage SIVD as compared to those with Alzheimer's disease (AD) and to older adults with normal cognition, and to explore the neural correlates of PM deficits.

**Method:** Patients with very-mild to mild dementia due to SIVD or AD and normal controls (NC) aged above 60 years were recruited. Seventy-three participants (20 SIVD, 22 AD, and 31 NC) underwent structural magnetic resonance imaging (MRI), cognitive screening tests, and a computerized PM test. Sixty-five of these participants (19 SIVD, 20 AD, and 26 NC) also received resting-state functional MRI.

**Results:** The group with SIVD had significantly fewer PM hits than the control group on both time-based and non-focal event-based PM tasks. Among patients in the very early stage, only those with SIVD but not AD performed significantly worse than the controls. Correlational analyses showed that non-focal event-based PM in SIVD was positively correlated with regional homogeneity in bilateral superior and middle frontal gyri, while time-based PM was not significantly associated with regional homogeneity in any of the regions of interest within the dorsal frontoparietal regions.

**Conclusions:** The findings of this study highlight the vulnerability of non-focal event-based PM to the disruption of regional functional connectivity in bilateral superior and middle frontal gyri in patients with SIVD.

## Introduction

Subcortical ischemic vascular disease accounts for a sizable proportion of patients hospitalized for cerebrovascular disease (CVD) (Chui, 2000). Unlike the typical abrupt onset of stroke, accumulating occult vascular insults, including lacunar infarction, white matter lesions, and enlarged perivascular spaces (Wardlaw et al., 2013) in subcortical ischemic vascular disease (SIVD), often result in an insidious and slowly progressive course that resembles Alzheimer's disease (AD) (O'Brien et al., 2003). As the evolving concept of vascular cognitive impairment (VCI) highlights the modifiable components of cognitive disorders (Roh and Lee, 2014), identifying the presence of vascular pathology is an important issue.

Executive dysfunction is the most commonly reported cognitive domain in SIVD, probably due to a vascular burden within the basal ganglia and white matter that interrupts the prefrontal-subcortical loop (Roman et al., 2002). Neuropsychological tests have been proven useful for assisting the differential diagnosis between vascular and AD pathology (Kertesz and Clydesdale, 1994; Graham et al., 2004), even in the very early stage (Ingles et al., 2007; Hsu et al., 2015). However, it remains unclear how SIVD pathology may affect a functionally important cognitive function termed prospective memory (PM), which also imposes a heavy load on frontal executive function (Martin et al., 2003; Burgess et al., 2011).

### Prospective Memory

Prospective memory is the remembering of an intended action to be performed under certain circumstances in the future (Kvavilashvili, 1998; McDaniel and Einstein, 2000), and it has been shown to have prominent functional relevance in normal aging and patient groups (Woods et al., 2012; Zogg et al., 2012). The unique prospective component of PM (i.e., the remembering to remember, rather than the content of the memory) (Dobbs and Rule, 1987), strongly relies on rostral prefrontal function (Cockburn, 1995; Burgess et al., 2003; Simons et al., 2006; Okuda et al., 2007). The executive component of PM varies according to the type of task. A focal event-based PM (EBPM) task, in which the ongoing task directs focal attention to the PM action cue, stimulates reliance on spontaneous retrieval processes. In contrast, a non-focal EBPM task, where the ongoing task does not direct attention to process the designated action cue, requires attention-demanding, strategic monitoring for successful performance (Einstein et al., 2005; Brewer et al., 2010; Scullin et al., 2010). In addition, another type of PM in which intention retrieval is triggered by a specific time (time-based prospective memory, TBPM) has been shown to require an intermediate amount of cognitive resources between that of focal and non-focal EBPM tasks, as it activates only transient monitoring (Oksanen et al., 2014). McBride and Flaherty (2020) also reported higher resource requirements as reflected by performance cost in non-focal EBPM tasks compared with the other two types of PM tasks.

### Prospective Memory in CVD

Few studies have examined PM in patients with CVD, and their findings have been inconclusive. A scoping review (Hogan et al., 2016) reported that previous studies of PM in patients who suffered stroke were limited by single-item measures. In addition, heterogeneity regarding the types of CVD may also have contributed to the inconsistent findings. To date, no previous study has focused on PM performance in patients with SIVD.

An additional research aim of the present study was to investigate whether there were differences in PM function between patients with AD and SIVD. Despite the growing body of PM research in AD and its risk groups (Driscoll et al., 2005; Jones et al., 2006; Costa et al., 2011; Hsu et al., 2015), few studies have compared the pattern of PM performance in patients with AD and SIVD, especially in the early stages. To the best of our knowledge, only the study by Livner et al. (2009) showed that both AD dementia and vascular dementia were impaired on a single-trial naturalistic PM task. As opposed to the signature failure of focal PM tasks in AD (McDaniel et al., 2011), we hypothesized that SIVD would have a greater effect on non-focal EBPM, followed by TBPM due to their disproportionate pathological loads on the frontosubcortical circuits.

### Neural Correlates of PM

Convergent evidence from neuroimaging studies has shown the importance of anterior frontal areas in PM (Burgess et al., 2001, 2003; Lamichhane et al., 2018). For example, Brodmann Area 10 (BA10) has been shown to be the central gateway to directing attention to either stimulus-oriented or stimulus-independent thoughts (Burgess et al., 2007). However, structural and functional connectivity has also been demonstrated to be critical to PM function in various disease entities (Hsu et al., 2019; Zhang et al., 2019; Zhu et al., 2019). A recent meta-analysis (Cona et al., 2015) further indicated that PM may rely on the frontoparietal network, which has also been implicated in executive control (Cole et al., 2014), attention function (Scolari et al., 2015), and even language processing (Fedorenko and Thompson-Schill, 2014). The anterior prefrontal cortex, dorsal frontal cortex, and dorsal parietal cortex within the dorsal frontoparietal network were considered to be particularly relevant to strategic monitoring processes, which are more highly required in non-focal EBPM tasks as compared with focal EBPM tasks. White matter lesions over the subcortical regions and downstream cortical dysfunction in SIVD may interrupt the links between the prefrontal cortex and subcortical nuclei (Roman et al., 2002), thereby compromising PM function. To the best of our knowledge, no previous study has investigated the relationships between PM, white matter burden, and functional connectivity in SIVD.

In this study, we used regional homogeneity (ReHo) from resting-state functional MRI (rs-fMRI) to assess regional functional connectivity by measuring the similarity of the time series of a given voxel to nearby voxels. As ReHo has been proven to be sensitive to neurodegenerative processes such as AD (Liu et al., 2008) and SIVD (Tu et al., 2020), we considered that ReHo may serve as an alternative method to detect regional anomalies that underlie PM failure.

Therefore, the present study aimed to investigate: (1) the clinical presentation of PM in SIVD; (2) differences in the performance of PM between SIVD and AD; and (3) the relationship between PM and cortical functional connectivity in SIVD. We hypothesized that patients in the SIVD group would perform worse than both those with AD and the normal controls (NC) on PM tasks, especially on non-focal EBPM tasks, and that both types of PM deficits would be associated with functional dysconnectivity in the dorsal frontoparietal areas.

## Materials and Methods

All participants entered the study after signing written informed consent. The study was approved by a local research ethics committee (REC106-48).

### Participants

Eligible participants were adults aged between 60 and 95 years who fulfilled the research criteria. A total of 73 participants (20 SIVD, 22 AD, and 31 NC) received a conventional MRI with Fazekas scores rated by the attending neurologist and completed neuropsychological examinations (Supplementary Table 1). Participants, 14 SIVD and 14 AD, with clinical dementia rating (CDR) 0.5 were considered to have very mild (or very early) dementia. Among them, eight participants did not complete the rs-fMRI survey, including two showing intolerance to prolonged scanning time and six showing poor fMRI imaging quality. In total, 65 participants (19 SIVD, 20 AD, and 26 NC) completed additional rs-fMRI examinations with acceptable image quality.

The inclusion criteria for the SIVD group were the following: (1) evidence of SIVD based on the criteria of Erkinjuntti et al. (2000); (2) Hachinski Ischemic Score (HIS) (Hachinski et al., 1975) ≥ 7; and (3) CDR (Morris, 1993) = 0.5–1. The inclusion criteria for the AD group were: (1) the National Institute on Aging-Alzheimer's Association Criteria (McKhann et al., 2011); (2) HIS ≤ 4; and (3) CDR = 0.5–1. The inclusion criteria for the NC group were (1) no evidence of cognitive deficits and (2) normal appearance on conventional MRI. The exclusion criteria were the same as in our previously published study protocol (Tu et al., 2017).

### Clinical Variables

Clinical variables, including systemic disease and psychotropic medications, were analyzed, using dummy coding on the basis of their possible impact on cognitive function and/or cerebral functional activity changes as reflected by rs-fMRI metrics. Systemic diseases, including hypertension, diabetes mellitus, hyperlipidemia, and coronary artery disease, were recorded as in our previously published study protocol (Tu et al., 2017). The use of psychotropics in the past 3 months was reviewed through electronic medical records, and was further categorized into acetylcholinesterase inhibitors, memantine, hypnotics, antidepressants, and antipsychotics.

### Cognitive Tests

General cognitive ability was measured, using the Taiwanese mini-mental state examination (MMSE) (Folstein et al., 1975; Shyu and Yip, 2001) and cognitive abilities screening instrument (CASI) (Lin et al., 2012). Dementia severity was rated, using the CDR (Morris, 1993), where CDR 1 indicates mild dementia and CDR 0.5 indicates very mild (or very early).

**Figure 1:**
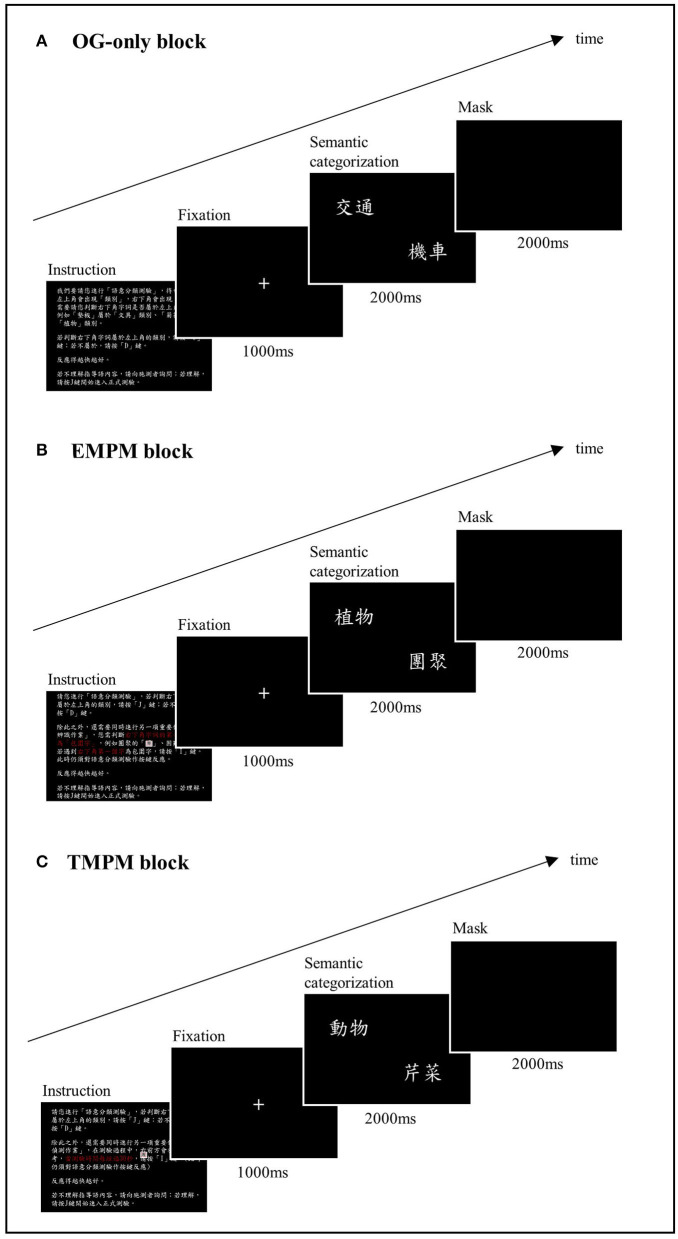
Schema of the computerized prospective memory task. **(A)** The OG-only block. The participants were instructed to decide if an exemplar presented on the bottom right corner of the screen belonged to the semantic category indicated on the left upper corner of the screen. **(B)** The EBPM block. The participants were instructed to perform an additional PM task while performing the ongoing task, in which they had to press a designated key when spotting a character that was perceptually enclosed. **(C)** The TBPM block. The participants were instructed to press a designated key at a 30 s interval from the beginning of the ongoing task.

Computerized PM tasks were constructed, using E-Prime software (Schneider et al., 2002). We followed the structure of the McDaniel–Einstein paradigm (Einstein and McDaniel, 1990) and adapted the task instructions from McDaniel et al. (2013) and Gonneaud et al. (2014). The task consisted of three blocks: an ongoing-only (OG) block, a non-focal EBPM block, and a TBPM block. The order of the blocks was counterbalanced across the participants. Practice trials were conducted with a different set of stimuli before the formal testing phase. The formal test started when the participants showed that they fully understood the task instructions. Figure 1 shows the schema of the PM task format. The instructions for each block are shown in Supplementary Material 2.

The ongoing task of the PM test was a semantic categorization task. In each trial, a word indicating a semantic category (e.g., “transportation”) was shown on the left upper corner of the screen, with another word indicating an exemplar (e.g., “car”) presented on the bottom right corner. The participant was instructed to press the “J” key (labeled as “Yes”) when the exemplar was judged to belong to the semantic category, or the “D” key (labeled as “No”) if not. Three lists of 78 pairings were used and counterbalanced across task blocks. Half of the pairings yielded a correct “yes” answer, and the other half yielded a correct “no” answer. The items were presented in random order. Each pair of stimuli appeared for 2 s, followed by masking for another 2 s. A correct response within these 4 s was recorded as a hit. The stimuli were relatively high-frequency words based on the Word List with Accumulated Word Frequency in Sinica Corpus (https://elearning.ling.sinica.edu.tw/eng_teaching.html). The percentage of correct responses (%) and reaction time (RT) in milliseconds (ms) were recorded.

In the EBPM block, the participants were asked to perform an additional PM task while performing the semantic categorization task. They had to press the “I” key (labeled in blue) when they detected a Chinese character that was perceptually enclosed, such as “團” or “困.” In this case, detection of the PM target demanded processing a perceptual feature that was different from the semantic processing during the ongoing task, constituting a *non-focal* EBPM task. Thirteen PM trials were randomly embedded in the ongoing task in this block. An EBPM *hit* was recorded if the participant pressed the blue key within 4 s after the EBPM target stimulus appeared. The RT in the EBPM block was measured by averaging the RT during the ongoing task trials that did not contain a PM target or an incorrect categorization response.

In the TBPM block, the participants were instructed to remember to press the “I” key (labeled in blue) at a 30 s interval from the beginning of the ongoing task. The experimenter placed an analog clock on the right front side of the participant to minimize the confounding of individual differences in time perception. The participants were told that if they forgot to press the target key, they could do so as soon as they remembered. There were a total of 13 potential hits for the time frame of this block. A PM hit was recorded if the participants pressed the blue key within a 2.5-s time frame before or after the correct timing. In our final analysis, we adjusted this time interval to between 2.5 and 5 s, and the results showed that a 2.5-s interval generated the most sensitive outcome variable. The RT in the TBPM block was measured by averaging the RT during ongoing task trials that did not contain a PM target or an incorrect categorization response.

In addition to the written instructions on the screen, a verbal explanation was given to ensure that the participants fully understood the task instructions. The participants were asked to summarize the task to the experimenter. The three response keys, “J,” “D,” and “I” were labeled as “Yes,” “No,” and in blue to facilitate comprehension of the task. The computerized PM task required about 20 min to complete. The outcome variables of this computerized task included correct response (hit rate, %) and RT (ms) in the OG, EBPM, and TBPM blocks.

### Neuroimaging Examinations

Magnetic resonance experiments were performed on a 3T MRI system (Discovery MR750; GE Medical System, Milwaukee, WI, USA) with an eight-channel phased-array head coil. Similar to the study by Tu et al. (2020), the imaging sequences included three-dimensional T1-weighted imaging (3D-T1), T2 fluid-attenuated inversion recovery imaging (T2-FLAIR), and rs-fMRI. For 3D-T1, the sequence parameters were an inversion time (TI) of 450 ms, flip angle of 12°, field-of-view of 240 mm, matrix size of 240 × 240, slice thickness (SL) of 1 mm, and 160 slices. For T2-FLAIR, the sequence parameters were repetition time of 12,000 ms, echo time of 120 ms, TI of 2,200 ms, FOV of 220 mm, matrix size of 384 × 224, slice thickness of 5 mm, and 21 slices. For rs-fMRI, the echo planar imaging (EPI) sequence parameters were a repetition time of 2,500 ms, echo time of 30 ms, flip angle of 90°, field-of-view of 200 mm, matrix size of 64 × 64, slice thickness of 3 mm, and 47 slices. The participants were asked to rest but not fall asleep during the examination. The magnetic resonance protocols took approximately 40 min to complete.

Periventricular and deep white matter hyperintensities (WMHs) from the T2-FLAIR sequences were rated, using the Fazekas Scale (Fazekas et al., 1993).

The resting state-fMRI data preprocessing procedures included realignment, slice timing adjustment by using windowed Fourier interpolation, co-registration between echo planar imaging, T1-weighted images, and spatial normalization of T1-weighted images to the Montreal Neurological Institute space (ICBM512 template), using Diffeomorphic Anatomical Registration Through Exponentiated Lie Algebra (DARTEL) (Ashburner, 2007). Framewise displacement was corrected, using the realign function of the Data Processing Assistant for Resting-State fMRI (DPARSF) software package (Yan and Zang, 2010), which involves realigning each image frame to the first frame, using the rigid body transformation. In addition, nuisance covariate regression was performed to reduce the confounding effects of spurious signals and head motion. The nuisance covariates include white matter signals, cerebrospinal fluid signals, and 24 head motion parameters (Friston et al., 1996). A band-pass filter was applied to the preprocessed time series, which removed the high-frequency noise (including signal spikes and physiological signals) and slow signal drifts. The pass band was set to 0.01–0.1 Hz based on the findings of the previous study (Biswal et al., 1995). A total of 144 time points were acquired for rs-fMRI. For fMRI data processing, the first 10 time points were discarded and not used for the following analysis (Yan and Zang, 2010). In the present study, the preprocessing step included image co-registration and normalization to the template, yielding a subtle intersubject difference of head size (or brain size) or white matter volume for those normalized T1 images (and co-registered functional images), which we then used to calculate the ReHo in the regions of interest (ROIs). Therefore, we did not include head size (or brain size) or white matter volume as the controlling factors in the statistical analysis. Image quality was examined by two of the authors (MCT and SYL). The exclusion of datasets with unacceptable image quality was determined by a panel discussion (MCT, LWK, and SYL).

Regional homogeneity was used as the index representative of cerebral functional connectivity. ReHo values were calculated according to a previous study (Zang et al., 2004). For each voxel, we used the neighboring 26 voxels to calculate its ReHo value. Subsequently, an isotropic Gaussian smoothing kernel (full width at half maximum = 4 mm) was applied to the estimated ReHo map in order to mitigate the effect of registration errors and intersubject variability (Mikl et al., 2008). All preprocessing procedures were performed using DPARSF toolbox (Yan and Zang, 2010). The ReHo measurements were initially analyzed at a voxel-wise level and then calculated within selected ROIs based on a previous study (Cona et al., 2015). The nomenclature of the ROIs in the current study was defined according to the automated anatomical labeling template (AAL; Tzourio-Mazoyer et al., 2002), including bilateral (i) superior frontal gyrus, medial portion (Frontal_Sup_Medial), (ii) superior frontal gyrus, dorsolateral portion, (Frontal_Sup), (iii) middle frontal gyrus (Frontal_Mid), and (iv) superior parietal gyrus (Parietal_Sup).

### Statistical Analysis

Statistical analyses were performed, using IBM SPSS Statistics 25 (Armonk, NY, USA). Between-group comparisons of demographic and clinical variables were performed, using the Chi-square tests or analysis of variance (ANOVA), followed by Scheffé or Dunnett's T3 *post-hoc* tests, where appropriate. Performance on the computerized PM test was compared between groups, using analysis of covariance (ANCOVA), controlling for potential covariates. *Post-hoc* tests were conducted with Bonferroni correction. Since we aimed to explore brain areas that were vulnerable to neurodegenerative processes in the early stage, we also conducted the same group mean comparison procedures among patients with only very early dementia (CDR = 0.5).

In order to examine the neural correlates of PM and the underlying cause of PM failure in SIVD, correlational analyses were performed to examine the relationship between PM hits and Fazekas scores, where partial correlation coefficients were used to account for potential confounding factors. The relationship between PM hits and ReHo parameters in the ROIs was then examined. A false discovery rate (FDR) was calculated for the correlational analysis between each type of PM and multiple ReHo values in each group.

## Results

There were significant group differences in age (*F* = 5.01, *p* = 0.009) and education (*F* = 3.61, *p* = 0.032) among all the participants (*n* = 73), and the NC group was younger and more educated (*p* < 0.05) (Table 1). All the participants were right-handed, and there was no significant difference in sex (χ^2^ = 0.42, *p* = 0.809). There were significant differences between groups in HIS (*F* = 211.85, *p* < 0.001) and Fazekas total scores (*F* = 73.67, *p* < 0.001), and the SIVD group had higher scores than the AD and NC groups (both *p* < 0.05). Regarding general cognitive function, there were significant group differences in MMSE (*F* = 27.08, *p* < 0.001) and CASI (*F* = 36.12, *p* < 0.001) scores. *Post-hoc* analyses showed that the two patient groups performed significantly worse than the NC group (both *p* < 0.05), but there was no significant difference between the SIVD and AD groups. Regarding comorbid systemic diseases, there were significant differences between groups in the frequency of CVD (*p* < 0.001), hypertension (*p* = 0.001), and coronary artery disease (*p* = 0*.0*01). There were also significant differences in the use of the following medications: antiplatelets (*p* = 0.001), antihypertensives (*p* = < 0.001), statins (*p* = 0.016), oral antidiabetic drugs (*p* = 0.020), acetylcholinesterase inhibitors (*p* < 0.001), memantine (*p* = 0.007), and hypnotics (*p* = 0.022).

**Table 1:** Basic information of the participants.

	**All participants**					**Patients with CDR** **=** **0.5 and NC**				
	**SIVD**	**AD**	**NC**	**ANOVA**		**SIVD^†^**	**AD^†^**	**NC**	**ANOVA**	
	**(***n*** = 20)**	**(***n*** = 22)**	**(***n*** = 31)**	***F/*** **χ^2^**	***p***	**(***n*** = 14)**	**(***n*** = 14)**	**(***n*** = 31)**	***F/*** **χ^2^**	***p***
Age (years)	70.40 ± 7.92	75.32 ± 6.95	67.26 ± 11.02	5.00	0.009^c^	68.71 ± 7.81	75.43 ± 7.31	67.26 ± 11.02	3.58	0.034^c^
Education (years)	6.80 ± 3.47	8.00 ± 4.63	10.03 ± 4.62	3.61	0.032^b^	7.57 ± 2.77	8.21 ± 4.85	10.03 ± 4.62	1.88	0.162
Sex (% of male)	60%	50%	55%	0.42	0.809	71%	43%	55%	2.35	0.309
Handedness (% of right-handedness)	100%	100%	100%	–	–	100%	100%	100%	–	–
Hachinski ischemic score	9.65 ± 1.76	1.50 ± 0.44	1.74 ± 1.32	211.85	<0.001^a,b^	9.86 ± 2.03	1.14 ± 1.29	1.74 ± 1.32	162.54	<0.001^a,b^
Fazekas scale										
Periventricular white matter	2.15 ± 0.49	1.00 ± 0.63	0.52 ± 0.63	46.61	<0.001^a,b,c^	2.07 ± 0.48	0.64 ± 0.18	0.52 ± 0.63	32.83	<0.001^a,b^
Deep white matter	2.25 ± 0.44	0.57 ± 0.60	0.45 ± 0.62	67.89	<0.001^a,b^	2.21 ± 0.43	0.54 ± 0.66	0.45 ± 0.62	45.84	<0.001^a,b^
Total score	4.40 ± 0.82	1.57 ± 0.93	0.97 ± 1.17	73.67	<0.001^a,b^	4.29 ± 0.73	1.46 ± 1.13	0.97 ± 1.17	47.58	<0.001^a,b^
Systemic diseases (% of presence)										
Cerebrovascular disease	45%	0%	6%	19.70	<0.001^a,b^	36%	0%	6%	10.37	0.006^a,b^
Hypertension	75%	32%	26%	13.27	0.001^a,b^	79%	21%	26%	13.46	0.001^a,b^
Diabetes mellitus	30%	14%	16%	1.05	0.354	21%	7%	16%	1.14	0.564
Hyperlipidemia	65%	45%	42%	1.38	0.258	64%	36%	42%	2.69	0.261
Chronic kidney disease	25%	14%	6%	3.54	0.171	29%	7%	6%	4.90	0.086
Coronary artery disease	25%	0%	0%	14.22	0.001^a,b^	29%	0%	0%	13.79	0.001^a,b^
Medication (% of use)										
Antiplatelet	80%	32%	32%	13.46	0.001^a,b^	86%	14%	32%	16.72	<0.001^a,b^
Anticoagulant	10%	0%	3%	2.77	0.251	14%	0%	3%	3.43	0.180
Anti-hypertensives	75%	36%	16%	17.87	<0.001^a,b^	79%	21%	16%	18.20	<0.001^a,b^
Statin	70%	45%	29%	8.24	0.016^b^	71%	36%	29%	7.37	0.025
Oral antidiabetic drug	40%	14%	10%	7.84	0.020^b^	29%	7%	10%	3.58	0.167
Acetylcholinesterase inhibitor	10%	68%	0%	36.21	<0.001^a,b^	0%	57%	0%	29.75	<0.001^a,c^
Memantine	0%	18%	0%	9.81	0.007^a,c^	0%	14%	0%	6.65	0.036^a,c^
Hypnotics	20%	45%	13%	7.66	0.022^c^	14%	43%	13%	5.76	0.056
Antidepressants	25%	32%	19%	1.08	0.583	21%	43%	19%	2.96	0.227
Antipsychotics	0%	5%	0%	2.35	0.309	0%	7%	0%	3.27	0.195
CASI	68.65 ± 13.54	71.18 ± 11.62	90.39 ± 5.63	36.12	<0.001^b,c^	74.86 ± 9.64	77.29 ± 7.010	90.39 ± 5.63	30.59	<0.001^b,c^
MMSE	22.10 ± 4.61	22.27 ± 4.026	28.19 ± 1.70	27.08	<0.001^b,c^	23.71 ± 4.01	24.29 ± 2.67	28.19 ± 1.70	18.81	<0.001^b,c^

*SIVD, subcortical ischemic vascular disease; AD, Alzheimer's disease; NC, normal controls; ANOVA, analysis of variance; CDR, Clinical Dementia Rating; CASI, Cognitive Abilities Screening Instrument; MMSE, Mini-Mental State Examination*.

† *Patients in very early stage (CDR = 0.5)*.

a *significant difference between the SIVD and AD groups*.

b *Significant difference between the SIVD and NC groups*.

c *Significant difference between the AD and NC groups by Scheffé or Dunnett's T3 tests (p < 0.05)*.

In comparisons of patients in the very early stage (CDR 0.5) with those in the NC group, there was a significant difference in age (*F* = 3.58, *p* = 0.034) but not in education (*F* = 1.88, *p* = 0.162) or sex (χ^2^ = 2.35, *p* = 0.309). The AD group was significantly older than the NC group (*p* < 0.05). There were also significant group differences in HIS (*F* = 162.54, *p* < 0.001) and Fazekas total scores (*F* = 47.58, *p* < 0.001), and the SIVD group had higher scores than the AD and NC groups (*p* < 0.05). Furthermore, group differences were found in CASI (*F* = 30.59, *p* < 0.001) and MMSE (*F* = 18.81, *p* < 0.001) scores, and the NC group performed significantly better than the AD and SIVD groups (*p* < 0.05).

### PM Performance

Regarding PM performance in all the participants (Table 2), age and education were chosen as covariates to adjust for the differences between groups (Table 1). There were significant between-groups differences in both EBPM (*F* = 9.60, *p* < 0.001, η_*p*_^2^ = 0.22) and TBPM (*F* = 8.82, *p* < 0.001, η_*p*_^2^ = 0.21) hit rates after controlling for age and education. *Post-hoc* analyses showed that both the SIVD (EBPM: *p* = 0.003; TBPM: *p* = 0.001) and AD (EBPM: *p* = 0.001; TBPM: *p* = 0.005) groups performed significantly worse than the NC group; however, there was no significant difference between the SIVD and AD groups. There was no significant group difference in RT in either the EBPM or TBPM blocks. In the OG block, there were significant between-groups differences in both correct response (*F* = 10.00, *p* < 0.001; η_*p*_^2^ = 0.23) and RT (*F* = 4.36, *p* = 0.017, η_*p*_^2^ = 0.11). *Post-hoc* analyses showed that both the SIVD (*p* < 0.001) and AD (*p* = 0.012) groups performed significantly worse than the NC group in the OG correct rate, and that the AD group had a significantly longer RT than the NC group (*p* = 0.017).

**Table 2:** Computerized PM task performance.

	**All participants**						**Patients with CDR** **=** **0.5 and NC**					
	**SIVD**	**AD**	**NC**	**ANCOVA^†^**			**SIVD†**	**AD†**	**NC**	**ANCOVA^‡^**		
	**(***n*** = 20)**	**(***n*** = 22)**	**(***n*** = 31)**	***F***	***P***	***η*** **^2^**	**(***n*** = 14)**	**(***n*** = 14)**	**(***n*** = 31)**	***F***	***P***	***η*** **^2^**
EBPM block												
PM hit (%)	31.2 ± 29.8	24.5 ± 30.2	67.7 ± 30.9	9.60	<0.001^a,b^	0.22	41.2 ± 29.0	36.3 ± 31.6	67.7 ± 30.9	4.60	0.014^a^	0.14
RT (ms)	2834.77 ± 546.49	2800.86 ± 570.84	2294.07 ± 622.40	3.03	0.054	0.08	2830.63 ± 562.44	2686.56 ± 598.32	2294.07 ± 622.40	3.95	0.025^a^	0.13
TBPM block												
PM hit (%)	38.1 ± 32.4	43.0 ± 42.7	78.4 ± 17.8	8.82	<0.001^a,b^	0.21	45.6 ± 31.4	60.4 ± 39.7	78.4 ± 17.8	6.62	0.003^a^	0.19
RT (ms)	2582.38 ± 566.05	2669.03 ± 557.56	2177.09 ± 599.37	1.93	0.153	0.05	2533.71 ± 597.20	2590.56 ± 595.21	2177.09 ± 599.37	1.75	0.183	0.06
OG block												
Correct (%)	64.9 ± 26.8	71.4 ± 25.1	94.2 ± 7.9	10.00	<0.001^a,b^	0.23	73.6 ± 23.9	77.0 ± 27.6	94.2 ± 7.9	6.40	0.003^a^	0.19
RT (ms)	2588.98 ± 568.28	2668.47 ± 615.73	2079.62 ± 565.70	4.36	0.017^b^	0.11	2502.39 ± 602.98	2574.08 ± 636.42	2079.62 ± 565.70	2.88	0.065	0.10

*SIVD, subcortical ischemic vascular disease; AD, Alzheimer's disease; NC, normal controls; ANCOVA, analysis of covariance; CDR, Clinical Dementia Rating; PM, prospective memory; EBPM, event-based prospective memory; TBPM, time-based prospective memory; RT, reaction time; OG, ongoing-only*.

† *Patients in the very early stage (CDR = 0.5)*.

‡ *Age and education as covariates*.

a *Significant difference between the SIVD and NC groups*.

b *Significant difference between the AD and NC groups by Bonferroni test (p < 0.05)*.

When inspecting only patients in the very early stage (CDR = 0.5; Table 2), there were group differences in the PM hit rate in both EBPM (*F* = 4.60, *p* = 0.014, η_*p*_^2^ = 0.14) and TBPM (*F* = 6.62, *p* = 0.003, η_*p*_^2^ = 0.19) blocks. *Post-hoc* analyses showed that there were only significant differences between the SIVD and NC groups (EBPM: *p* = 0.027; TBPM: *p* = 0.002). In addition, there was a significant group difference in RT in the EBPM block (*F* = 3.95, *p* = 0.025, η_*p*_^2^ = 0.13), implying a significantly larger performance cost with the additional EBPM intention in the SIVD group than in the other groups (*p* = 0.02).

### Correlations Between PM and Fazekas Scores

In order to examine the neural correlates of PM deficit, correlational analyses were first performed to examine the relationship between PM hits and Fazekas scores in each group. We preliminarily examined the relationship between PM and Fazekas scores with potential confounding factors in each group, including demographic variables (i.e., age, education, and sex), vascular risk factors (e.g., hypertension, diabetes mellitus, hyperlipidemia, and coronary artery disease), and medication (e.g., antiplatelet, anticoagulant, antihypertensives, statins, oral antidiabetic drug, insulin, acetylcholinesterase inhibitors, memantine, hypnotics, antidepressants, and antipsychotics), using Pearson or point-biserial correlation coefficients, where appropriate. The relationships between the PM hit rate and Fazekas scores were then examined after accounting for the effects of the identified confounding factors.

In the SIVD group, age was correlated with Fazekas total scores (*r* = 0.539, *p* = 0.017), the EBPM hit rate (*r* = −0.56, *p* = 0.014) and the TBPM hit rate (*r* = −0.57, *p* = 0.011). Thus, partial correlation coefficients between Fazekas scores and PM performance were calculated, accounting for the effect of age. The results showed that there were no significant correlations between EBPM and Fazekas scores (periventricular: *r* = −0.30, *p* = 0.223; deep white matter: *r* = −0.04, *p* = 0.891; total score: *r* = −0.19, *p* = 0.452) or between TBPM and Fazekas scores (periventricular: *r* = −0.12, *p* = 0.632; deep white matter: *r* = 0.10, *p* = 0.694; total score: *r* = −0.009, *p* = 0.971) within the SIVD group.

There were also no significant relationships between Fazekas scores and the PM hit rate in either the AD (*p* = 0.160–0.896) or the NC group (*p* = 0.259–0.735). *Post-hoc* analyses among all the participants showed that there were significant correlations between the TBPM hit rate and Fazekas scores in the periventricular white matter (*r* = −0.40, *p* = 0*.0*01), deep white matter (*r* = −0.24, *p* = 0.05) and total scores (*r* = −0.33, *p* = 0.005) and between the EBPM hit rate and Fazekas scores in periventricular areas (*r* = −0.27, *p* = 0.02).

### Correlations Between PM and ReHo

Regarding the association between ReHo and PM performance, we first examined the relationships between these two variables with potential confounding factors in each group, including demographic variables (i.e., age, education, and sex), vascular risk factors (e.g., hypertension, diabetes mellitus, hyperlipidemia, and coronary artery disease), and medication (e.g., antiplatelets, anticoagulants, antihypertensives, statins, oral antidiabetic drugs, insulin, acetylcholinesterase inhibitors, memantine, hypnotics, antidepressants, and antipsychotics), using Pearson or point-biserial correlation coefficients, where appropriate. The relationships between the PM hit rate and ReHo parameters were then examined after accounting for the effects of the identified confounding factors.

**Figure 2:**
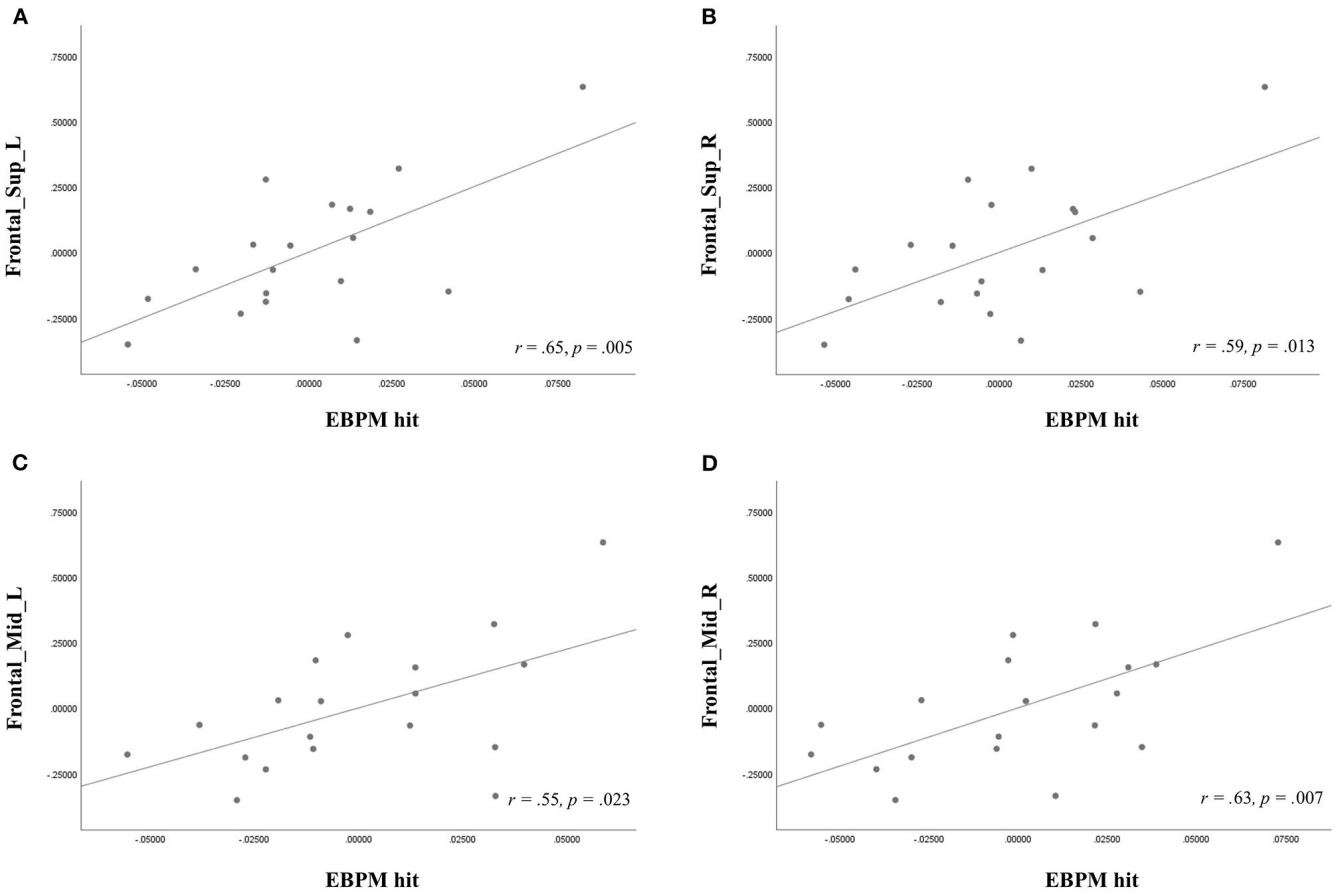
Partial correlation analysis, showing the relationship of event-based prospective memory (EBPM) hit with regional homogeneity (ReHo) in the **(A)** left superior frontal gyri, dorsolateral portion (Frontal_Sup_L), **(B)** right superior frontal gyri, dorsolateral portion (Frontal_Sup_R), **(C)** left middle frontal gyrus (Frontal_Mid_L), and **(D)** right middle frontal gyrus (Frontal_Mid_R) after controlling for age and antihypertensives use in the subcortical ischemic vascular disease group.

In the SIVD group, age was correlated with the EBPM hit rate (*r* = −0.56, *p* = 0.014) and the TBPM hit rate (*r* = −0.57, *p* = 0.011), while the use of antihypertensives was related to ReHo (observed in the Frontal_Mid_R, *r* = 0.47, *p* = 0.041). Therefore, these two factors were used as covariates in the subsequent analysis. Fazekas scores were not entered into the equation due to considerations of multicollinearity as they were already correlated with age. A partial correlation analysis controlling for age and the use of antihypertensives (Table 3 and Figure 2) showed that there were significant correlations between the EBPM hit rate and ReHo in the Frontal_Sup_L (*r* = 0.65, *p* = 0.005), Frontal_Sup_R (*r* = 0.59, *p* = 0.013), Frontal_Mid_L (*r* = 0.55, *p* = 0.023), and Frontal_Mid_R (*r* = 0.63, *p* = 0.007). However, there were no significant relationships between the TBPM hit rate and ReHo in any of the ROIs. The aforementioned correlation results in the SIVD group remained significant after adjustment by FDR. An analysis of patients with very early dementia (CDR 0.5) showed a similar pattern of correlations, as can be seen in Supplementary Table 2.

**Table 3:** Partial correlation between PM performance and ReHo in ROIs controlling for confounding variables.

	**EBPM hit**				**TBPM hit**			
	**Left hemispheres**		**Right hemispheres**		**Left hemispheres**		**Right hemispheres**	
	***r***	***p***	***r***	***p***	***r***	***p***	***r***	***p***
The SIVD group*								
Frontal_Sup	**0.65**	**0.005**	**0.59**	**0.013**	0.29	0.263	0.38	0.136
Frontal_Sup_Medial	0.45	0.069	0.39	0.122	0.14	0.605	−0.12	0.646
Frontal_Mid	**0.55**	**0.023**	**0.63**	**0.007**	0.32	0.204	0.42	0.091
Parietal_Sup	0.4	0.116	0.26	0.319	0.04	0.877	0.28	0.272
*The AD group* ^†^								
Frontal_Sup	−0.33	0.213	−0.31	0.241	−0.08	0.781	−0.06	0.816
Frontal_Sup_Medial	−0.38	0.144	−0.50	0.046	−0.20	0.467	−0.27	0.320
Frontal_Mid	−0.13	0.637	−0.33	0.213	−0.07	0.804	−0.20	0.463
Parietal_Sup	−0.13	0.624	−0.37	0.158	−0.31	0.242	−0.39	0.136
*The NC group* ^‡^								
Frontal_Sup	0.11	0.626	0.00	0.998	0.09	0.681	0.17	0.449
Frontal_Sup_Medial	0.16	0.454	0.10	0.647	0.25	0.249	0.22	0.302
Frontal_Mid	0.12	0.591	0.05	0.821	0.21	0.332	0.27	0.221
Parietal_Sup	0.01	0.973	0.03	0.899	0.02	0.921	−0.06	0.769

*SIVD, subcortical ischemic vascular disease; AD, Alzheimer's disease; NC, normal controls; PM, prospective memory; EBPM, event-based prospective memory; TBPM, time-based prospective memory; ReHo, regional homogeneity; ROIs, regions of interest; Frontal_Sup_Medial, superior frontal gyrus, medial portion; Frontal_Sup, superior frontal gyrus, dorsolateral portion; Frontal_Mid, middle frontal gyrus; Parietal_Sup, superior parietal gyrus. Bold font indicates significant results after controlling for the false discovery rate (q = 0.05)*.

* *Controlling for age and use of antihypertensives*.

† *Controlling for Fazekas scores in the deep white matter, the use of antiplatelets, statins, and hypnotics*.

‡ *Controlling for age, education, and use of antidepressants*.

In the AD group, significant correlations were found between ReHo values and Fazekas scores in the deep white matter, and the use of antiplatelets, statins, and hypnotics (*p* < 0.05). After controlling for these variables, a significant relationship was found between the EBPM hit rate and ReHo in Frontal_Sup_Medial_R (*r* = −0.50, *p* = 0.046), but not between the TBPM hit rate and any ReHo parameters (*p* = 0.136–0.816). The aforementioned correlation result in the AD group was not significant after being adjusted by FDR.

In the NC group, significant correlations were found between the EBPM hit rate and age (*r* = −0.40, *p* = 0.042) and education (*r* = 0.59, *p* = 0.002), and between ReHo (observed in Frontal_Mid_R) and the use of antidepressants (*r* = 0.53, *p* = 0.005). After controlling for these three factors, no significant relationships were found between any ReHo values and the EBPM hit rate (*p* = 0.454–0.998) or the TBPM hit rate (*p* = 0.221–0.921).

## Discussion

The aim of this study was to examine PM performance in patients with SIVD and explore neural substrates relevant to PM failure. The results showed that the patients with SIVD performed worse on PM tasks than the healthy aging controls, in particular during the very early stage compared with the patients with AD. Although EBPM failure was associated with subcortical WMHs when considering all the participants, PM deficits were only related to changes in functional connectivity within the bilateral superior and middle frontal gyri in the SIVD group. Using ReHo metrics, cortical dysfunction involving regions far beyond the WMHs on structural MRI was identified.

### PM Performances

Performance deficits were observed in both non-focal EBPM and TBPM in the patients with SIVD, which is consistent with the findings reported by Man, Chan, and Yip (Man et al., 2015) and partially in line with those reported by Kim et al. (2009). However, our findings are different from the study by Brooks et al. (2004), who reported only EBPM impairment but not TBPM impairment. This discrepancy may be due to differences in the time interval used in the two studies, with 30 s in our study and 5 min in that of Brooks et al. Albeit understudied, there are preliminary results showing that TBPM with different time intervals may be associated with disparate underlying cognitive processes (Nigro et al., 2002). Our findings indicated that a shorter time interval may be more sensitive to PM deficits, at least in patients with SIVD.

We also observed an additional performance cost in the very early SIVD group during the EBPM task compared with the NC group, as reflected by the prolonged RT during the ongoing trials. Performance cost has been reported to indicate the additional cognitive resources needed to hold a PM intention in mind. This phenomenon has been found to be more prominent in healthy older adults than in healthy young adults, indicating either an increased need of attention resources for strategic monitoring (Smith, 2003) or a more conservative attention allocation policy (Heathcote et al., 2015). The resource-consuming monitoring process is considered to be related to frontal executive processes (McDaniel et al., 2011, 2013) and more likely to be observed in non-focal EBPM tasks (Scullin et al., 2010), as can be seen in the present study.

In this study, we also attempted to examine the potential use of PM tests in differentiating patients with SIVD from those with AD. Although there was no significant performance difference between the SIVD and AD groups, we found that PM deficits were only observed in the SIVD group but not the AD group in the very early stage (CDR = 0.5). This is in line with our hypothesis that SIVD patients are prone to exhibit non-focal EBPM and TBPM failure, together with other frontal executive deficits.

Theoretically, successful PM performance requires functional integrity in the rostral prefrontal cortex (Burgess et al., 2007). However, PM is also a complex psychological process that requires the synergistic operation of multiple brain areas/neural circuits (Cona et al., 2015). This is likely the reason why there was a lack of significant difference in PM performance between the SIVD and AD groups, since they both suffered from spreading pathological changes in the brain as the disease progressed. In addition to the frontosubcortical dysregulation that is well-recognized in small vessel diseases (Roman et al., 2002; Roh and Lee, 2014), previous studies have shown that amyloidosis and tauopathy in AD can induce myelin breakdown and lead to disconnection in not only medial temporal limbic connections (Kantarci et al., 2017) but also in fibers connecting the frontal lobes (Allen et al., 2007). Therefore, the lack of differential value of PM tests may be because PM relies on a widespread neural network, and because there are overlapping brain areas, which are interrupted by both AD and SIVD pathologies.

### Neural Correlates of PM

In this study, we also aimed to investigate the neural basis of PM deficits in SIVD. Previous studies have indicated a negative effect of WMHs on cognitive function, especially in the attention and executive function domains (Bolandzadeh et al., 2012; Kloppenborg et al., 2014). Some studies have further reported a dose-dependent, causal link in the associations between large confluent WMHs and cognitive impairment (Prins and Scheltens, 2015). Whether WMHs in different brain regions exert disparate effects on different cognitive domains remains a thriving research topic (Bolandzadeh et al., 2012). To date, little is known about the impact of spatial WMH distribution on PM. Our findings showed that PM deficits were associated with both periventricular and deep WMHs in all the participants, while the relationship was not significant when inspecting only the SIVD or AD groups. This finding is partially consistent with the results of Holz et al. (2017), in which a trend of correlation was only found between PM and WMHs in a group of people with MCI and mild AD (*p* = 0.052). Therefore, the impact of WMHs on PM may be threshold-sensitive.

In the present study, we also identified cerebral functional connectivity related to PM in SIVD. Correlations between non-focal EBPM hits and ReHo were observed among the patients with SIVD in the bilateral middle frontal gyri and the dorsolateral part of the superior frontal gyri. Despite the lack of a significant association with the superior medial frontal gyrus, the correlation pattern remained consistent with our hypothesis that the disruption of neural substrates responsible for strategic monitoring in SIVD would compromise the performance on non-focal EBPM and TBPM. The dorsal prefrontal cortex is considered to be essential to the top-down control of maintaining task goals (Cona et al., 2015). According to the gateway hypothesis (Burgess et al., 2003, 2007), the processing in the anterior prefrontal cortex regarding PM concerns suppressing and maintaining spontaneous thoughts. The lateral but not medial portion of the prefrontal cortex is particularly important for maintaining this internally generated cognition, and it was found to be associated with PM performance in our patients with SIVD.

On the other hand, we did not find any associations between TBPM with either WMHs or ReHo in SIVD. Previous studies on neural correlates of PM have concentrated mostly on EBPM rather than TBPM. Some studies have indicated that TBPM may share neural substrates with EBPM in frontoparietal areas (Gonneaud et al., 2014; Oksanen et al., 2014). However, we failed to confirm this finding. Although TBPM is generally considered to require more self-initiation and resource-consuming processes, it is likely that our task design, which required the participants to press a key within a set time interval, led to a relatively habitual action. Further studies are needed to examine the relationship between TBPM and the ventral frontoparietal network that supports spontaneous retrieval processes.

Unlike the positive correlation observed in the SIVD group, the results revealed a marginal negative correlation between the EBPM hit and ReHo in the right superior medial frontal gyrus in the AD group. A similar spatial pattern was observed in a previous study where patients with SIVD showed reduced ReHo in the right middle frontal gyrus and left anterior cingulate gyrus, while patients with AD had higher ReHo in the right orbital frontal areas (Tu et al., 2020). The reason for this opposite direction of correlations is unknown. Given that neuronal correlates responsible for PM were found to vary across each PM stage (Cona et al., 2015), inter-ROIs interactions during the PM processes could potentially contribute to the disparate correlation patterns observed in SIVD and AD. From a viewpoint that incorporates both neuroimaging and psychological perspectives, an association between cognitive impairment and the disconnection process has been evidenced in cerebral small vessel disease (Schulz et al., 2021). On the other hand, increased ReHo in several brain areas has been found in AD (Wang et al., 2007), which was considered to reflect a compensatory mechanism when additional cognitive resources were recruited to fulfill task demands.

### Limitations

There are several limitations to the present study. First, the sample size was relatively small and the control group was demographically unmatched. Nevertheless, potential confounders were controlled through statistical procedures, and the effect size was provided for reference. Second, the PM tasks required literacy, which restricted the number of eligible participants in our setting. Third, we did not conduct amyloid scanning to exclude the possibility of mixed etiologies in our SIVD groups. Moreover, despite the evident close relationship between Fazekas scores and WMH volume assessments (Valdés Hernández Mdel et al., 2013), Fazekas scores may be suboptimal in providing detailed region-specific information. Future studies are recommended with more advanced techniques to quantify WMHs. In addition, the AAL template was built based on anatomical information (Tzourio-Mazoyer et al., 2002). Although the parcellations of AAL are not functionally distinct, the AAL template has been widely used in functional connectivity analysis, and it has been shown to be useful for identifying several kinds of neurological diseases, including Alzheimer's disease, mild cognitive impairment, and Parkinson's disease (Wee et al., 2012; Khazaee et al., 2015; Liu et al., 2019). Nevertheless, brain atlases built based on resting-state functional connectivity may be beneficial in further investigations (Power et al., 2011; Gordon et al., 2016; Schaefer et al., 2018). Lastly, the main findings were based on correlations of PM performances with ReHo over selected ROIs in the SIVD group, but not on group differences in ROIs.

In conclusion, in favor of *a priori* knowledge of structural and functional changes in SIVD, the present study is the first to identify an association between PM and frontal dysconnectivity, using ReHo, and indicates the possibility that cerebral disconnection processes are responsible for PM deficits in SIVD.

## Data Availability Statement

The raw data supporting the conclusions of this article will be made available by the authors, without undue reservation.

## Ethics Statement

The present study was reviewed and approved by Taichung Tzu Chi Hospital Research Ethics Committee. The participants provided their written informed consent to participate in this study.

## Author Contributions

X-MZ: neuropsychological test assessment, data analysis, interpretation, and drafting the article. L-WK: study concept and revising the article. S-YL: neuroimaging processing, data analysis, and revising the article. J-JY: neuroimaging processing and data analysis. M-CT: patient recruitment, clinical examination, and revising the article. Y-HH: study concept and design, data analysis, interpretation, drafting, and revising the article. All authors contributed to the article and approved the submitted version.

## Conflict of Interest

The authors declare that the research was conducted in the absence of any commercial or financial relationships that could be construed as a potential conflict of interest.

## Publisher's Note

All claims expressed in this article are solely those of the authors and do not necessarily represent those of their affiliated organizations, or those of the publisher, the editors and the reviewers. Any product that may be evaluated in this article, or claim that may be made by its manufacturer, is not guaranteed or endorsed by the publisher.
