# Supplementary material for: Prospective Memory and Regional Functional Connectivity in Subcortical Ischemic Vascular Disease
Source: Front Aging Neurosci. 2021 Aug 20;13:686040. doi: 10.3389/fnagi.2021.686040 (PMC8417716; doi:10.3389/fnagi.2021.686040)
Supplement: Supplementary file 1 [file Data_Sheet_1.docx]

Supplementary Material 1

| **Table S1. Participants' performance on detailed neuropsychological examinations** | | | | | | | | | | | |
| --- | --- | --- | --- | --- | --- | --- | --- | --- | --- | --- | --- |
|  | **All participants** | | | | |  | **Patients with CDR = .05 and NC** | | | | |
|  | **SIVD** | **AD** | **NC** | **ANOVA** | |  | **SIVD^†^** | **AD^†^** | **NC** | **ANOVA** | |
|  | **(*n* = 20)** | **(*n* = 22)** | **(*n* = 31)** | ***F*** | ***P*** |  | **(*n* = 14)** | **(*n* = 14)** | **(*n* = 31)** | ***F*** | ***P*** |
| **CVVLT** |  |  |  |  |  |  |  |  |  |  |  |
| Immediate recall | 18.25±6.05 | 16.55±4.71 | 25.13±4.72 | 21.18 | <0.001 ^b, c^ |  | 19.36±6.66 | 18.57±3.69 | 25.13±4.72 | 11.08 | <0.001 ^b, c^ |
| 30-second short delay recall | 4.30±2.30 | 3.23±2.16 | 6.97±1.52 | 25.96 | <0.001 ^b, c^ |  | 4.93±2.43 | 4.36±1.74 | 6.97±1.52 | 12.37 | <0.001 ^b, c^ |
| 10-minute long delay recall | 2.65±2.98 | 2.36±2.42 | 6.42±2.08 | 22.84 | <0.001 ^b, c^ |  | 3.70±3.27 | 3.21±2.42 | 6.42±2.08 | 12.84 | <0.001 ^b, c^ |
| 10-minute cued recall | 5.35±2.56 | 3.09±2.37 | 7.23±1.94 | 21.68 | <0.001 ^a, b, c^ |  | 6.07±2.30 | 3.93±2.37 | 7.23±1.94 | 11.52 | <0.001 ^a, c^ |
| 10-minute recognition | 7.50±1.91 | 7.45±1.47 | 8.42±0.89 | 4.03 | 0.022 ^c^ |  | 8.14±1.41 | 7.86±1.03 | 8.42±0.89 | 1.4 | 0.254 |
| **CTT** |  |  |  |  |  |  |  |  |  |  |  |
| CTT1 (*ms*) | 197.80±123.35 | 140.91±110.98 | 72.29±39.78 | 11.6 | <0.001 ^b, c^ |  | 129.93±48.58 | 111.29±50.58 | 72.29±39.78 | 9.24 | <0.001 ^b, c^ |
| CTT2 (*ms*) | 396.75±194.88 | 260.00±151.13 | 151.29±70.09 | 19.12 | <0.001^a,b,c^ |  | 313.50±139.26 | 236.62±112.63 | 151.29±70.09 | 13.26 | <0.001 ^b, c^ |
| **TFAB** |  |  |  |  |  |  |  |  |  |  |  |
| Total score | 9.30±2.89 | 10.36±2.79 | 13.65±1.94 | 21.58 | <0.001 ^b, c^ |  | 10.29±2.52 | 10.93±2.89 | 13.65±1.94 | 12.74 | <0.001 ^b, c^ |
| **Digit Span (WAIS-III)** |  |  |  |  |  |  |  |  |  |  |  |
| Forward score | 9.30±2.45 | 9.55±2.11 | 11.94±2.17 | 11.29 | <0.001 ^b, c^ |  | 9.50±2.24 | 9.93±1.82 | 11.94±2.17 | 8.26 | 0.001 ^b, c^ |
| Backward score | 3.80±1.64 | 4.59±1.56 | 6.81±2.73 | 13.6 | <0.001 ^b, c^ |  | 4.29±1.73 | 4.93±1.69 | 6.81±2.73 | 6.94 | 0.002 ^b, c^ |
| **SDMT** |  |  |  |  |  |  |  |  |  |  |  |
| Correct | 12.70±7.78 | 19.09±9.46 | 34.00±14.53 | 23.09 | <0.001 ^b, c^ |  | 15.71±7.35 | 21.79±8.82 | 34.00±14.53 | 12.8 | <0.001 ^b, c^ |
| Error | 0.90±1.25 | 1.27±2.12 | 0.84±1.10 | 0.57 | 0.566 |  | 0.71±1.14 | 0.64±0.63 | 0.84±1.09 | 0.19 | 0.82 |
| **^†^**Patients in very early stage (CDR = 0.5); CVVLT: Chinese Version Verbal Learning Test (Chang et al., 2010); CTT: Color Trails Test (D'Elia et al., 1996); TFAB: Taiwanese Frontal Assessment Battery (Wang et al., 2016); WAIS-III: Wechsler Adults Intelligence Scale - the Third Edition (Wechsler, 2002); SDMT: Symbol Digit Modality Test (Smith, 1982); ^a^ Significant difference (*p* < .05) between SIVD and AD groups by the Bonferroni test; ^b^ Significant difference (*p* < .05) between SIVD and NC groups by the Bonferroni test; ^c^ Significant difference (*p* < .05) between AD and NC groups by the Bonferroni test. | | | | | | | | | | | |

Supplementary Material 2

# Instruction for the OG block:

*“In the following semantic categorization task, there will be a word indicating a ‘category’ on the left upper corner; and another word indicating a ‘exemplar’ on the right bottom corner of the screen. Please determine if the exemplar belongs to the category. For example, ‘Pad’ belongs to the category ‘stationary’; and ‘chrysanthemum’ belongs to the category ‘plants’. If the exemplar belongs to the category, please press the ‘J’ key; if not, please press the ‘D’ key. Please respond as fast as you can. If you do not understand the instruction, please consult the experimenter; if you do, please press ‘J’ to start the experiment.”*

# Instruction for the EBPM block:

*“In the following semantic categorization task, there will be a word indicating a ‘category’ on the left upper corner; and another word indicating an ‘exemplar’ on the right bottom corner of the screen. Please determine if the exemplar belongs to the category. For example, ‘Pad’ belongs to the category ‘stationary’; and ‘chrysanthemum’ belongs to the category ‘plants’. If the exemplar belongs to the category, please press the ‘J’ key; if not, please press the ‘D’ key. In addition, remember to do another important ‘perceptual identification task’. You will have to decide if the first character of the exemplar is an ‘enclosed’ Chinese word. If so, please press the “I” key. Meanwhile, you still need to continue the semantic categorization task. Please respond as fast as you can. If you do not understand the instruction, please consult the experimenter; if you do, please press ‘J’ to start the experiment.”*

# Instruction for the EBPM block:

*“In the following semantic categorization task, there will be a word indicating a ‘category’ on the left upper corner; and another word indicating a ‘exemplar’ on the right bottom corner of the screen. Please determine if the exemplar belongs to the category. For example, ‘Pad’ belongs to the category ‘stationary’; and ‘chrysanthemum’ belongs to the category ‘plants’. If the exemplar belongs to the category, please press the ‘J’ key; if not, please press the ‘D’ key. In addition, remember to do another important ‘time detection task’. There will be a clock placed in the right front side of you. Please press the “I” key every 30 seconds. Meanwhile, you still need to continue the semantic categorization task. Please respond as fast as you can. If you do not understand the instruction, please consult the experimenter; if you do, please press ‘J’ to start the experiment.”*

Supplementary Material 3

| **Table S2. Partial correlation between PM performance and ReHo in ROIs controlling for confounding variables in patients with CDR 0.5** | | | | | | | | |
| --- | --- | --- | --- | --- | --- | --- | --- | --- |
|  | EBPM hit | | | | TBPM hit | | | |
|  | Left hemispheres | | Right hemispheres | | Left hemispheres | | Right hemispheres | |
|  | *r* | *p* | *r* | *p* | *r* | *p* | *r* | *p* |
| *The SIVD group (CDR 0.5) ^*^* |  |  |  |  |  |  |  |  |
| Frontal_Sup | 0.79 | **0.002** | 0.77 | **0.003** | 0.39 | 0.210 | 0.53 | 0.076 |
| Frontal_Sup_Medial | 0.61 | **0.035** | 0.64 | **0.024** | 0.32 | 0.309 | 0.05 | 0.870 |
| Frontal_Mid | 0.62 | **0.031** | 0.72 | **0.009** | 0.41 | 0.188 | 0.50 | 0.097 |
| Parietal_Sup | 0.73 | **0.008** | 0.60 | **0.041** | 0.18 | 0.573 | 0.28 | 0.376 |
|  |  |  |  |  |  |  |  |  |
| *The AD group (CDR 0.5)* ^†^ |  |  |  |  |  |  |  |  |
| Frontal_Sup | -0.37 | 0.369 | -0.14 | 0.747 | -0.02 | 0.959 | -0.02 | 0.969 |
| Frontal_Sup_Medial | -0.24 | 0.568 | -0.78 | 0.023 | -0.07 | 0.860 | -0.46 | 0.249 |
| Frontal_Mid | -0.05 | 0.908 | -0.13 | 0.764 | 0.11 | 0.792 | 0.03 | 0.949 |
| Parietal_Sup | 0.36 | 0.377 | -0.39 | 0.344 | 0.39 | 0.338 | -0.25 | 0.555 |
| PM: prospective memory; SIVD: subcortical ischemic vascular disease; AD: Alzheimer’s disease; EBPM: event-based prospective memory; TBPM: time-based prospective memory; ReHo: regional homogeneity; ROIs: regions of interest; Frontal_Sup_Medial: superior frontal gyrus, medial portion; Frontal_Sup: superior frontal gyrus, dorsolateral portion; Frontal_Mid: middle frontal gyrus; Parietal_Sup: superior parietal gyrus. Bold font indicates significant results after controlling for false discovery rate (*q* = .05); *Controlling for age and use of anti-hypertensives, †Controlling for Fazekas scores in the deep white matter, the use of antiplatelets, statins and hypnotics. | | | | | | | | |

**References**

Chang, C. C., Kramer, J. H., Lin, K. N., Chang, W. N., Wang, Y. L., Huang, C. W., Lin, Y. T., Chen, C., & Wang, P. N. (2010, Mar). Validating the Chinese version of the Verbal Learning Test for screening Alzheimer's disease. *Journal of the International Neuropsychological Society, 16*(2), 244-251. <https://doi.org/10.1017/S1355617709991184>

D'Elia, L. F., Satz, P., Uchiyama, C., & White, T. (1996). *Color Trails Test Professional Manual*. Psychological Assessment Resources. <https://doi.org/doi:10.1097/00002093-200204000-00003>

Smith, A. (1982). *Symbol Digits Modalities Test*. Western Psychological Services.

Wang, T. L., Hung, Y. H., & Yang, C. C. (2016). Psychometric Properties of the Taiwanese (Traditional Chinese) Version of the Frontal Assessment Battery: A Preliminary Study. *Appl Neuropsychol Adult, 23*(1), 11-20. <https://doi.org/10.1080/23279095.2014.995792>

Wechsler, D. (2002). *Wechsler Adult Intelligence Scale - Third edition (WAIS-III): Technical and interpretative manual for Taiwan*. Chinese Behavioral Science Corp.
